# Imaging biomarkers for detecting *IDH* mutations and monitoring response to novel targeted therapies: Current insights and future perspectives

**DOI:** 10.1093/neuonc/noag028

**Published:** 2026-02-16

**Authors:** Archith Rajan, Felipe Rosero Castro, Laiz Laura de Godoy, Mauro Hanaoka, Sevcan Turk, Lisa Desiderio, Roger Stupp, Suyash Mohan, Sanjeev Chawla

**Affiliations:** Department of Radiology, Perelman School of Medicine, University of Pennsylvania, Philadelphia, Pennsylvania, USA; Department of Radiology, Perelman School of Medicine, University of Pennsylvania, Philadelphia, Pennsylvania, USA; Department of Radiology, Perelman School of Medicine, University of Pennsylvania, Philadelphia, Pennsylvania, USA; Department of Radiology, Perelman School of Medicine, University of Pennsylvania, Philadelphia, Pennsylvania, USA; Department of Radiology, Perelman School of Medicine, University of Pennsylvania, Philadelphia, Pennsylvania, USA; Department of Radiology, Perelman School of Medicine, University of Pennsylvania, Philadelphia, Pennsylvania, USA; Division of Hematology and Oncology, Department of Medicine, Feinberg School of Medicine, Northwestern University, Chicago, Illinois, USA; Departments of Neurological Surgery and Neurology, Feinberg School of Medicine, Northwestern University, Chicago, Illinois, USA; Department of Radiology, Perelman School of Medicine, University of Pennsylvania, Philadelphia, Pennsylvania, USA; Department of Radiology, Perelman School of Medicine, University of Pennsylvania, Philadelphia, Pennsylvania, USA

## Abstract

Isocitrate dehydrogenase (*IDH*) mutant gliomas represent a unique molecular subset of gliomas with distinct metabolic and microstructural characteristics. The recent approval of targeted *IDH* inhibitors marks a significant advancement in glioma therapy, thereby necessitating robust, quantitative methods for noninvasive assessment of treatment response. This review provides an overview of advanced multiparametric imaging techniques—including proton MR spectroscopy, diffusion and perfusion MRI, amide proton transfer imaging, and amino acid positron emission tomography imaging—and their role in detecting *IDH-*mutations and monitoring therapeutic response to *IDH* inhibitors. Special emphasis is placed on metabolic imaging of the oncometabolite D-2-hydroxyglutarate (2-HG), a hallmark signature of *IDH-*mutant gliomas, and how its quantification serves as a surrogate biomarker for diagnosis and treatment monitoring. We also highlight the potential of advanced diffusion MRI-based models, which capture microstructural alterations beyond conventional apparent diffusion coefficient metrics. Some limitations of these techniques in clinical translation are also considered, along with future directions to integrate them into prospective clinical trials.

Key Points

- Advanced imaging biomarkers enable noninvasive monitoring of isocitrate dehydrogenase (*IDH*) inhibitor response.
- Quantification of D-2-hydroxyglutarate (2-HG) via proton MR spectroscopy (^1^H-MRS) is central to diagnosis and treatment tracking.
- Multiparametric MRI/MRS provide complementary insights into therapy-induced alterations in the tumor microenvironment.

Gliomas, harboring isocitrate dehydrogenase (*IDH*) mutations, constitute a biologically distinct subset of primary brain tumors with characteristic molecular and metabolic features that influence prognosis and response to therapy. The standard-of-care treatment approach for *IDH-*mutant gliomas includes maximal safe resection, followed by either surveillance (watchful waiting) or additional treatment with radiation therapy and/or chemotherapy based on patient age, glioma grade, and extent of resection^1^^,^^2^; however, treatment-related toxicities can be consequential in this typically young, functionally intact population with long survival. The recent approval of *IDH*-targeted inhibitors (eg, vorasidenib) highlights the need for developing reliable, noninvasive methods to determine *IDH*-mutational status and quantify early therapeutic response.

Mutant *IDH* (mIDH) drives accumulation of an oncometabolite, D-2-hydroxyglutarate (2-HG), which perturbs epigenetic regulation and immune function and provides a compelling metabolic target for imaging-based detection and pharmacodynamic monitoring.

Neuroimaging and quantitative multiparametric techniques that integrate diverse data from several imaging modalities provide a comprehensive assessment of underlying pathophysiology and disease progression, thereby improving our ability to precisely monitor response to therapy.^3^ In addition to conventional magnetic resonance imaging (MRI) sequences, advanced modalities such as diffusion MRI (dMRI), perfusion and permeability MRI, proton MR spectroscopy (^1^H-MRS), amide proton transfer (APT), and positron emission tomography (PET) imaging techniques offer valuable insights into the tumor physiology and metabolic alterations at the cellular level. These advanced imaging methods have the potential to serve as reliable biomarkers, capable of detecting early biological changes prior to structural alterations becoming visible on conventional neuroimaging.

This review provides an overview of the recently approved novel targeted therapies (*IDH*-inhibitors) for treating *IDH-*mutant gliomas. We summarize “state-of-the-art” quantitative multiparametric imaging methods for (1) detecting *IDH-*mutations and (2) monitor response to targeted therapies. We emphasize metabolic imaging of 2-HG with ¹H-MRS, recent advances in quantitative diffusion and perfusion MRI, APT and amino-acid PET, and practical considerations for their clinical implementations, while outlining priorities for prospective validation and integration into trials and routine care.

## *IDH-*Mutations and *IDH* Inhibitors

### *IDH*-Mutation and Gliomagenesis

**Figure 1.**
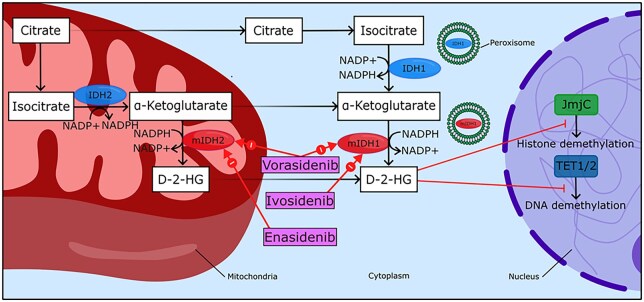
Glioma cell *IDH-*mutants and their inhibitors. Isocitrate is converted to α-ketoglutarate by wild-type isocitrate dehydrogenase 1 (*IDH1*) and *IDH2* (*blue ovals*) in a reaction that generates NADPH from NADP+. While *IDH1* is found in the cytosol and peroxisomes, *IDH2* is present in the mitochondrial matrix. In glioma cells with mutant *IDH1* and *IDH2* (mIDH1/2), α-ketoglutarate is converted to D-2-hydroxyglutarate (D-2-HG), oxidizing NADPH to NADP+ in the process. By competitively inhibiting the activity of α-ketoglutarate-dependent demethylases such as TET1/2 DNA demethylase and JmjC histone demethylases, elevated 2-HG promotes DNA and histone hypermethylation. Vorasidenib is a dual mIDH1 and mIDH2 inhibitor, Ivosidenib inhibits mIDH1, and Enasidenib inhibits mIDH2, reducing 2-HG production.

The cytosolic *IDH1* and the mitochondrial *IDH2* constitute key enzymes responsible for oxidizing isocitrate into α-ketoglutarate (α-KG) and producing NADPH, a molecule important in regulating oxidative stress.^4^^,^^5^ Mutant *IDH* converts α-KG to 2-HG in a reaction consuming NADPH.^6^ At high concentrations, 2-HG leads to increased methylation of DNA and histones, which has a varied and not fully elucidated effect on the expression of tumor suppressor genes and oncogenes.^4^ Elevated 2-HG may furthermore contribute to oncogenesis via 2-HG-mediated immunosuppressive effects (Figure 1). Although *IDH-*mutations are frequently found in glial neoplasms in humans, it is worth noting that studies introducing *IDH-*mutations into transgenic mice did not observe the development of gliomas in mouse models. It is believed that this may be because *IDH-*mutations may indirectly facilitate oncogenesis in gliomas via the suppressive effect of 2-HG on cellular differentiation by extending the cell cycle and inducing oxidative stress, which allows for the introduction of more potent oncogenic mutations, such as TP53 mutations and 1p19q codeletions, which are strongly associated with *IDH-*mutations in astrocytomas and oligodendrogliomas, respectively.^5^^,^^7^^,^^8^ Notably, studies using ^1^H-MRS have shown higher levels of 2-HG in in vivo *IDH2*-mutant gliomas compared to those with mutations to *IDH1*.^9^^,^^10^ Another study using ^1^H-MRS further showed that higher concentrations of 2-HG in *IDH-*mutant gliomas correlated with higher glioma grade but found no association between 2-HG concentrations and histologic subtype of glioma per WHO 2016 classification guidelines, including oligodendroglioma, astrocytoma, mixed glioma, and secondary glioblastoma.^11^^,^^12^

### *IDH* Inhibitors and Their Action Mechanism

Since the 2009 discovery of *IDH-*mutations in gliomas and their likely role in oncogenesis, there has been continued interest in developing therapeutic strategies to inhibit mIDH to improve outcomes in these patients.^13^^,^^14^ Among the first *IDH* inhibitors to be tested in clinical trials were ivosidenib, a mutant-*IDH1* inhibitor, and vorasidenib, a dual inhibitor of mutant-*IDH1* and mutant-*IDH2*. Initial phase I trials of both drugs showed generally favorable safety profiles, with preliminary response rates appearing to favor vorasidenib, especially in non-enhancing gliomas.^15^^,^^16^ A perioperative phase I trial was further conducted to establish the effects of both drugs in human *IDH-*mutant gliomas, ultimately showing that 2-HG concentrations in tumor cells extracted after treatment with higher doses of either ivosidenib or vorasidenib were reduced by more than 90% compared to untreated controls, a finding consistent with preclinical studies.^17^ Vorasidenib showed better brain penetration, a slightly higher objective response rate, and more consistent 2-HG suppression, which led to its selection for a subsequent phase III trial.^17^ The INDIGO (INvestigating vorasiDenIb in GliOma: NCT04164901) Phase 3 trial enrolled 331 patients with residual or recurrent Grade 2 *IDH*-mutant gliomas (no prior radiotherapy or chemotherapy), randomized to daily vorasidenib (40 mg) or placebo, demonstrating significantly improved imaging-based progression-free survival (PFS) and delaying subsequent therapies.^18^ The trial found imaging-based progression in 28% of patients receiving vorasidenib and in 54% of patients in the placebo arm, with median PFS being significantly longer in the group receiving vorasidenib (27.7 months vs 11.1 months) and with a safety profile consistent with prior trials.^4^^,^^16^ In light of these results, vorasidenib received FDA approval on August 6, 2024, for the treatment of *IDH-*mutant grade-2 gliomas following surgery.^18^ Moving forward, an additional 6 months of follow-up data have reaffirmed the previously reported statistically significant and clinically meaningful improvements in PFS and time to next intervention associated with vorasidenib.^19^ These benefits were consistent across patients, regardless of their baseline tumor volume. A notable observation was that patients’ tumor volumes tended to increase before starting vorasidenib treatment but subsequently decreased once treatment was initiated. This reduction in tumor volume was consistently observed even in patients who crossed over from the placebo arm to receive vorasidenib.^20^ These data provide further evidence of the drug’s sustained efficacy in the patient population.

## Need for Developing Imaging Biomarkers for Identification of *IDH-*Mutational Status and Response Assessment in Gliomas

While immunohistochemical analyses and exomic sequencing are considered as gold standards for determining *IDH-*mutational status and monitoring treatment response to targeted therapies in gliomas,^12^^,^^13^ their practical application is limited by several factors, including tissue heterogeneity, partial tumor sampling, variations in antigen expression levels, and diagnostic procedure.^21^^,^^22^ Additionally, the eloquent locations of these gliomas may limit the neurosurgical interventions. These limitations can lead to inaccurate determination of *IDH-*mutational status, especially when relying solely on a single biopsy sample or when antigen expression is not uniform throughout the tumor. Moreover, repeated and multiple invasive sampling is not feasible for monitoring the therapeutic potential of targeted agents. Therefore, there is an unmet need to develop noninvasive biomarkers for the identification of *IDH-*mutational status and provide an objective prediction of treatment response to targeted therapies.

### Conventional MR Imaging

**Figure 2.**
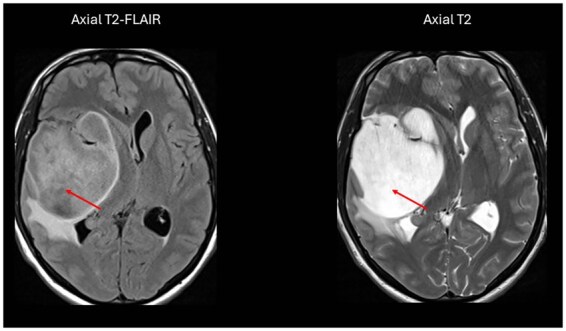
T2/FLAIR mismatch sign is visible in an *IDH-*mutant astrocytoma.

On conventional neuroimaging, some distinctive features have been proposed to identify *IDH-*mutant gliomas. These include well-defined tumor borders and no/minimal contrast enhancement within the tumor beds. Additionally, the “T2-FLAIR mismatch sign” has been found to be a noninvasive marker for the identification of *IDH-*mutant and 1p/19q-intact astrocytomas with high specificity but low sensitivity.^23^^,^^24^ This sign is characterized by complete or near-complete and homogeneous hyperintense signal intensity on T2-weighted images within a lesion, and hypointense signal intensity on T2-FLAIR images except for a hyperintense peripheral rim (Figure 2). This mismatch sign reflects underlying microcystic changes and low tumor cellularity and is generally accompanied by increased apparent diffusion coefficient (ADC) values (facilitated diffusion), homogeneous hypointense signal intensity on pre-contrast T1-weighted images, homogeneous hypodensity on CT imaging, and low cerebral blood volume (CBV). The clinical relevance of this mismatch sign has been confirmed in real-world patient cohorts,^23^ and large-scale imaging-genomic analyses such as those from TCGA/TCIA.^25^ While “T2-FLAIR mismatch sign” is generally considered highly specific for identifying *IDH-*mutant gliomas, false-positive results can occur especially in tumors showing contrast enhancement. Moreover, subcortical astrocytomas may appear to have hypointense regions within an otherwise hyperintense tumor on T2-weighted images due to the presence of interposed gray matter, potentially leading to misinterpretation. In such cases, obtaining images in different anatomical planes may be beneficial for better evaluation. As morphologic imaging characteristics are usually evaluated qualitatively and are observer-dependent, the assessment of these features may be subjective and have considerable user bias.

Emerging evidence also highlights the limitations of low-grade glioma-response assessment in neuro-oncology (LGG-RANO) criteria^26^ in reliable assessment of *IDH* inhibitors such as ivosidenib and vorasidenib in nonenhancing gliomas.^27^^,^^28^ Because these tumors are evaluated primarily through T2/FLAIR signal abnormalities, interpretation is confounded by non-tumor factors—including postoperative change, edema, ischemia, radiotherapy effects, and reactive gliosis—that can mimic or obscure true tumor response. The bidimensional (2D) measurements required by LGG-RANO criteria perform poorly in infiltrative, irregular lesions with ill-defined margins and show only modest interobserver agreement. These criteria also fail to capture the slow and often subtle radiographic changes characteristic of *IDH*-mutant gliomas, leading to underestimation of meaningful but small reductions in tumor size and making minor response difficult to assess consistently. Additionally, 2D LGG-RANO criteria may diverge substantially from volumetric measurements, which often detect progression earlier and with less variability. As a result, LGG-RANO criteria may misrepresent treatment effects of targeted agents whose primary impact may be tumor stabilization or a reduction in growth kinetics rather than large radiographic shrinkage.^29^

Thus, there is an urgent need for the development of more effective and alternative neuroimaging techniques for accurate response assessment. Contrary to conventional anatomic MR imaging methods, metabolic and physiologic imaging techniques such as proton MR spectroscopy, diffusion, and perfusion MR imaging are more sensitive to biophysical processes within the tissues and hence provide more comprehensive information about the tumor microenvironment, including changes in tumor metabolic landscape, cellularity, angiogenesis, hemodynamics, and vascular permeability. These techniques have been successfully integrated with standard MR imaging acquisition protocols at many institutions around the world. Readers are referred to excellent review articles about these methods with specific applications to neuro-oncology.^30–33^

In the subsequent sections, an overview of the potential utility of metabolic, physiologic MR imaging as well as PET imaging techniques in the identification of *IDH-*mutant gliomas and evaluation of treatment response to novel *IDH* inhibitors in gliomas is provided. Additionally, a summary of key features, potential challenges, and future directions for various imaging techniques used to assess therapeutic response to *IDH* inhibitors in gliomas is presented in Table 1.

**Table 1.** Key features of various therapeutic assessment techniques for *IDH* inhibitors in gliomas.

Technique	General features	Potential strengths	Potential challenges	Future directions in therapeutic assessment to *IDH* inhibitors	Trends in MRI/MRS parameters with *IDH* inhibitors
^1^H-MRS (TE = 97 ms)	A spin-echo sequence with an optimized echo time of 97 ms enables the detection of 2-HG peak at 2.25 ppm.	Easy to implement on a clinical 3T MRI scanner. Both single voxel and multivoxel sequences can be acquired in reasonable time frame with high data quality.	Extensive overlap of 2-HG resonances with those from other metabolites in the spectral region of 2-3 ppm. False positive rates in the range of 20%-30%.	Establishment of a reliable Cramér-Rao lower bound (CLRB) cutoff value for the detection of 2-HG.	**↓2-HG** (*IDH*-inhibition) **↑Glutamate** (metabolic reprogramming)
2D-COSY	Dispersion of multiplet structure of scalar (J)-coupled spin systems into a second spectral dimension. Unlikely possibility that 2 metabolites would share identical chemical shifts in 2 dimensions.	Unambiguous identification of overlapping resonances. Potential application in identification of Glu, Gln, GABA, 2-HG, PC, GPC, PE, GPE.	Long acquisition times, limited spatial coverage, and lack of readily available postprocessing tools.	Developing and implementing faster, multivoxel sequences along with a prior-knowledge-based fitting algorithm for data processing.	**↓2-HG** (*IDH*-inhibition) **↑Glutamate** (metabolic reprogramming)
Diffusion MRI	Assess microscopic, thermally induced translational (Brownian) motion of water molecules in biological tissues.	ADC/MD measures the magnitude of water motion and is inversely proportional to tumor cellular density. FA signifies the degree of diffusion anisotropy and is sensitive to white matter integrity and coherent organization of tumor cells.	Nonspecificity of the common DWI/DTI metrics to the underlying biological phenomenon. Inherently noisy technique prone to eddy current and susceptibility-induced artifacts.	Multiparametric longitudinal assessment with perfusion imaging in larger cohorts. Correlation with 2-HG concentration. Incorporation of advanced multicompartment model-based metrics beyond the ADC in clinically feasible acquisition times.	**↓FA/Kurtosis** **↑MD/ADC** (↓cellularity)
DSC-perfusion	Susceptibility-induced signal loss on dynamic T2*-weighted sequences after the administration of an intravenous GBCA.	Relative cerebral blood volume (rCBV) is a biomarker of angiogenesis and assesses tumor vascularity.	CBV maps are generally sensitive to susceptibility artifacts and these maps may be degraded by the presence of intra/peritumoral hemorrhages.	Delineation of distinct “tumor habitats” (different spatial regions within tumors based on tumor vascularity) using clustering methods to address the issue of intratumoral heterogeneity.	**↓rCBV** (↓vascularity/angiogenesis)
DCE-Perfusion	Involves acquisition of dynamic T1-weighted images before, during, and after the injection of an intravenous GBCA. Measures T1 changes in tissues over time after bolus administration.	*K^trans^* is a putative biomarker of angiogenesis; ve shows an inverse correlation with cellularity, and vp reflects angiogenic activity in tumors.	High sensitivity to the selected pharmacokinetic (PK) model and to AIF definition. In the routine clinical workflow, a fixed baseline T1 value is generally used instead of T1 mapping because of limited scan time.	Compressed sensing allows for faster acquisition of under-sampled k-space data, facilitating faster image reconstruction without significant loss of spatial resolution. Development of more sophisticated PK models (moving beyond extended Toft’s model) for DCE-MRI data analysis in clinical settings.	**↓*K^trans^*** **↓v_p_** (↓vascularity/angiogenesis)
APTw Imaging	Based on chemical exchange saturation transfer (CEST) for detecting and quantifying mobile proteins and peptides.	Quantitative mapping of proteins/peptides within the tumor microenvironment with higher spatial resolution. Higher protein concentration increases APT signal.	Lengthy acquisition time especially for 3-D sequences, higher RF power dissipation. Prone to artifacts due to B0 and B1 field inhomogeneities.	Optimization of pulse sequence and acquisition parameters, development of automated pipelines for image reconstruction, motion correction, and APT parameter mapping to streamline the analysis process.	**↓ APTw signal** (↓mobile peptides concentration)
Amino-acid-based PET	Tracers are characterized by high tumor-to-brain contrast based on their relatively high specificity for neoplastic cells and low accumulation in normal brain tissues.	Better delineation of glioma margins and differentiation of post-therapeutic changes (pseudo-progression, reactive changes, or radionecrosis) from true tumor progression, based on standard SUV or TBR-based cutoff values	Expensive and variably available tracers and PET/CT or PET/MRI systems, ionizing radiation exposure, requirement for specialized detectors and sophisticated attenuation correction and image reconstruction pipelines.	Develop high-resolution PET/MRI scanners, enhanced image reconstruction algorithms, and kinetic modeling for data processing. Improved tracer design.	**↓TBR_mean_ (≥ 10%)** **↓TBR_max_ (≥ 30%)** **↓metabolic tumor volume (≥ 40%)**

Abbreviations: 2-HG, 2-hydroxyglutarate; ADC, apparent diffusion coefficient; AIF, arterial input function; APT, amide proton transfer; CBV, cerebral blood volume; DCE, dynamic contrast enhanced; DSC, dynamic susceptibility contrast; DTI, diffusion tensor imaging; DWI, diffusion weighted imaging; FA, fractional anisotropy; GABA, gamma amino butyric acid; GBCA, gadolinium based contrast agent; Gln, glutamine; Glu, glutamate; GPC, glycerophosphocholine; GPE, glycerophosphoethanolamine; *K^trans^*, volume transfer constant; MD, mean diffusivity; PC, phosphocholine; PE, phosphoethanolamine; SUV, standardized uptake value; TBR, tumor to brain ratio; ve, extravascular extracellular space volume fraction; vp, plasma volume fraction.

### Magnetic Resonance Spectroscopy

Proton magnetic resonance spectroscopy (^1^H-MRS) provides a noninvasive biochemical profile useful for diagnosis, planning treatment strategies, and assessing treatment response in gliomas.^34–36^ An alteration in tumor metabolism leads to the accumulation of 2-HG, an oncometabolite, and a putative biomarker for identifying *IDH-*mutant gliomas.^6^^,^^37^ The detection of 2-HG in a brain tumor unambiguously confirms an *IDH-*mutational status as 2-HG is absent in normal, healthy brain cells.

The noninvasive detection of 2-HG on conventional ^1^H-MRS sequences is challenging due to the extensive overlap of its resonances with those from metabolites such as N-acetyl aspartate (NAA), glutamate (Glu), glutamine (Gln), gamma-aminobutyric acid (GABA), and lipids. Reliable in vivo detection and quantification of 2-HG typically requires the use of sophisticated ^1^H-MRS acquisition and postprocessing techniques. A standard point resolved spectroscopy (PRESS) sequence (TE = 30 ms) in combination with a commercially available spectral fitting algorithm (LCModel software^38^) led to a 26% false positive detection rate in *IDH*-wild-type gliomas because of severe spectral overlap between the 2-HG resonance (2.25 ppm) and other metabolites.

**Figure 3.**
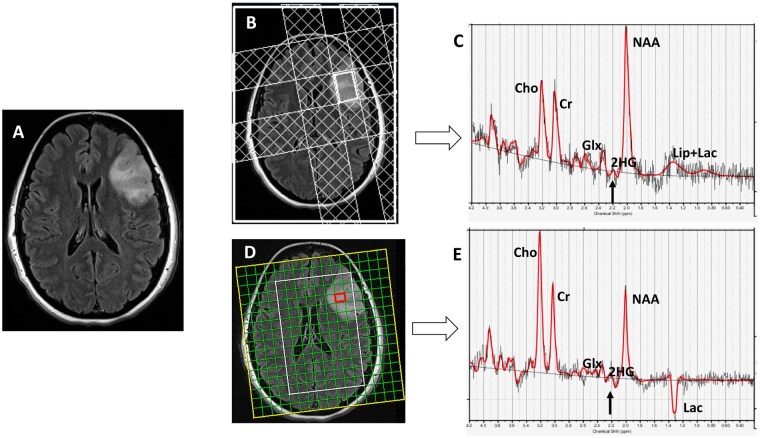
A patient with *IDH-*mutant anaplastic astrocytoma. (A) T2-FLAIR image shows a left frontal lobe mass. (B) Four outer-volume saturation slabs are placed outside the single voxel to suppress contamination from lipid signals arising from the scalp and bone marrow. (C) Spectra demonstrate elevated Cho/Cr (0.63; CRLB = 2%) and metabolic levels of 2-HG (2-HG/Cr = 0.4; CRLB = 33%; black arrow). (D) ^1^H-MRSI grid overlaid on T2-FLAIR image showing different voxels from tumor. (E) Spectra from a red voxel within the mass demonstrate elevated Cho/Cr (0.84; CRLB = 3%) and metabolic levels of 2-HG (2-HG/Cr = 0.49; CRLB = 36%, black arrow). Histopathological and immunohistochemical analyses were consistent with grade-3 astrocytoma with positive *IDH1* mutational status. CRLB, Cramer-Rao Lower Bound. Red lines: fitting spectra. Black lines: real spectra. Reprinted with permission from ref.^42^

In a seminal study, Choi et al. conducted several quantum mechanical simulation experiments and proposed to use an echo time (TE) of 97 ms in a PRESS sequence for the detection of 2-HG signals from gliomas undergoing scanning on 3T MR systems. The investigators of this study reported 100% sensitivity and specificity for detecting 2-HG in gliomas.^10^ Some other studies have also demonstrated the potential utility of ^1^H-MRS with TE = 97 ms in detecting and quantifying 2-HG in gliomas.^39–41^ Collectively, these studies have reported variable concentrations of 2-HG (5-35 mM) in *IDH-*mutant gliomas with detection accuracies varying between 53.85% and 94.12%. Moreover, increasing the sampled voxel size, particularly for tumors larger than 3.4 cm^3^ has been shown to improve the accuracy of 2-HG signal detection. In a recent study,^42^ our group prospectively analyzed the clinical utility of single voxel and single slice multivoxel ^1^H-MRS using an optimized TE of 97 ms in assessing *IDH-*mutational status by detecting the characteristic resonances of 2-HG in patients presenting with newly diagnosed infiltrative gliomas and suspected neoplastic progression (Figure 3). We observed that mean concentration and standard errors of 2-HG and metabolite ratio of 2-HG/Cr in *IDH-*mutant cases were 5.24 ± 1.59 mM and 0.55 ± 0.08, respectively. We also observed that ^1^H-MRS could identify *IDH-*mutant gliomas with high accuracy (79%), sensitivity (80%), and specificity (77%). Additionally, in line with our hypotheses and prior studies,^43^^,^^44^ the Glx (glutamate + glutamine)/Cr was found to be significantly decreased in *IDH-*mutant gliomas in our study. These results were supported by an earlier study reporting that glutamate acts as a main source of carbons for 2-HG in mIDH glioma cells^6^ and glutamate levels become depleted by the enzymatic activity of glutamate dehydrogenase so as to replenish α-KG lost in the conversion to 2-HG by the IDH enzyme.^45^ Moreover, metabolomic analyses using glioma cell lines and surgical specimens have reported that glutaminolysis serves as a key compensatory pathway to maintain metabolic homeostasis in *IDH-*mutant gliomas. As a consequence, glutamate levels are significantly reduced in *IDH-*mutant gliomas in comparison to *IDH-*wild-type gliomas.^46^ There have been continuing efforts to develop and refine ^1^H-MRS sequences specifically designed to detect 2-HG signals. In this direction, an optimized semi-LASER ^1^H-MRS sequence using a longer TE (110 ms) was proposed^47^ offering high sensitivity and localization for in vivo detection of 2-HG on 3T clinical MRI scanners. In a related study, the investigators used a triple refocusing sequence with a TE of 137 ms for improved detection of 2-HG by successfully suppressing the confounding signals from Glu, Gln, and GABA.^48^ Collectively, these technical improvements promise a more robust assignment of 2-HG metabolite for the identification of *IDH-*mutant gliomas and monitoring treatment response to targeted therapies.

Despite initial promising findings, all these studies employed single voxel and/or single slice multivoxel ^1^H-MRS sequences, which are constrained by limited spatial coverage. In contrast, a high-resolution whole-brain ^1^H-MRS technique has been developed for identification and quantification of 2-HG from the whole volume of *IDH-*mutant gliomas.^49^ On a similar line, another study demonstrated significantly higher Cho/Cr and Cho/NAA ratios in *IDH-*mutant gliomas than in *IDH-*wild-type counterparts using whole-brain echoplanar spectroscopic imaging (EPSI).^50^ Taking together, these findings provide a strong push to use the whole-brain ^1^H-MRS sequences for the noninvasive quantification and assessment of metabolite alterations in gliomas.

Despite the success of ^1^H-MRS methods in research, their integration into clinical workflows for the reliable and reproducible detection and quantification of 2HG remains hindered by significant technical variability, and analytic complexity across institutions and platforms. To bridge this gap and facilitate widespread clinical adoption, standardization, and building consensus on the use of both ^1^H-MRS sequences and acquisition parameters as well as processing tools are required. Additional improvement in this field requires greater data sharing and conduction of large multi-institutional and validation studies.

**Figure 4.**
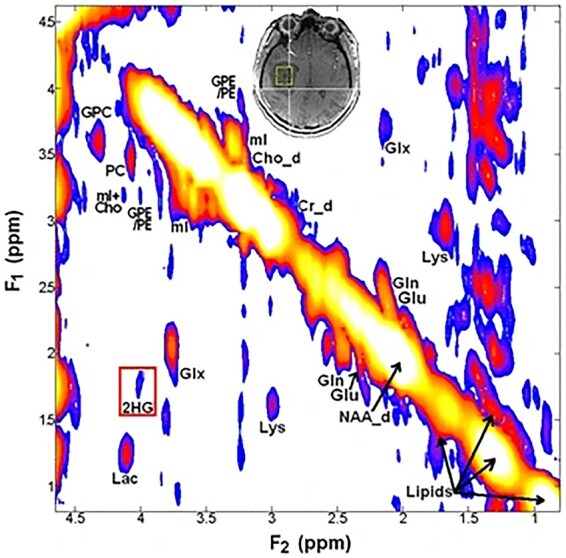
2-D COSY spectrum from an *IDH-*mutant glioma shows a cross peak (*F*2, *F*1 = 4.002, 1.9 ppm) corresponding to 2-HG and other clinically relevant metabolites, which may not be well identified on conventional 1D-^1^H-MRS. Reprinted with permission from ref.^52^

An alternative strategy to detect 2-HG is to use 2-dimensional correlated spectroscopy (2D-COSY). This sequence is superior to conventional 1D-MRS because it spreads the signals across 2 frequency dimensions, creating distinct “cross-peaks” that better resolve potentially overlapping resonances. Ultra-high-field 7T MRI provides a higher signal-to-noise ratio (SNR) and improved chemical shift dispersion compared to conventional 3T clinical systems. Leveraging these advantages, we employed 7T 2D-COSY sequence and reported our initial findings for identifying *IDH-*mutant gliomas by unambiguously detecting resonances of 2-HG besides describing other clinically relevant neuro-metabolites (Figure 4).^52^ Despite its promise, the 2D-COSY sequence is limited by some shortcomings including a relatively longer acquisition time than conventional 1D-^1^H-MRS sequences. To address this issue, techniques like compressed sensing^53^ or sparse sampling may be used. Furthermore, accuracy and reliability of metabolite quantification in the 2D-COSY sequence could be improved by leveraging a prior-knowledge based fitting algorithm such as ProFit^54^ rather than using the traditional peak integration method that may introduce subjective bias.

After initially using 2-HG primarily as a diagnostic biomarker, the focus has since shifted towards using 2-HG as a therapeutic biomarker for monitoring treatment response in *IDH-*mutant gliomas. In this direction, a prospective longitudinal clinical study reported a significant decline in 2-HG/Cr levels at 1-3 months after the end of chemoradiation therapy relative to baseline.^55^ This seminal study indicates that quantitative measurement of 2-HG over time may be used for early response assessment in clinical trials of therapies targeting *IDH-*mutant gliomas, however, with a caveat. In ^1^H-MRS, accurately placing the spectroscopy voxel is crucial for reliably monitoring the treatment response, as it ensures that the same anatomical location is probed each time, which is essential for detecting subtle changes in metabolite concentrations. Nonetheless, achieving consistent and precise placement of voxels at different time points can be challenging due to patient motion and variations in tumor characteristics (eg, size, shape, and spatial heterogeneity).

### Diffusion MR Imaging

The biophysical mechanism of diffusion-weighted imaging (DWI) is based on random, microscopic, thermally induced translational motion of water molecules in biological tissues. The magnitude of this random movement is described by its ADC. Several biochemical properties including temperature, viscosity, cellular density, cell membranes, intracellular organelles, and macromolecules impact the ADC values of biological tissues.^56^

Some studies have reported the potential utility of ADC values in differentiating *IDH-*mutant from *IDH-*wild-type gliomas. Collectively, these studies have reported higher ADC in *IDH-*mutant gliomas in comparison with their *IDH-*wild-type counterparts.^57–59^ This was primarily attributed to the *IDH-*wild-type gliomas typically being more aggressive in nature and hence exhibiting relatively greater cellularity, in turn resulting in greater restricted diffusion, and an associated decrease in ADCs.^60^ The relative ADC, minimum, maximum, and other percentiles of ADCs computed within the enhancing and non-enhancing tumor ROIs have all been used to classify *IDH-*mutant from *IDH-*wild-type gliomas, with varying degrees of accuracies, sensitivities, and specificities.^61–64^  The potential of ADC in classification of *IDH-*mutant subtypes based on 1p/19q codeletion, which is characteristic of oligodendrogliomas, and O^6^-methylguanine-DNA methyltransferase (MGMT) promoter methylation has also been explored.^59^^,^^65^^,^^66^ It was demonstrated that advanced MRI metrics, particularly diffusion and perfusion parameters, can reliably predict molecular characteristics of oligodendrogliomas. Tumors with *IDH*-mutation and 1p/19q codeletion status consistently exhibited lower mean ADC values, distinct histogram skewness, and unique perfusion profiles, reflecting higher cellularity and vascular differences compared to non-codeleted tumors.^59^^,^^65^^,^^66^

Recently, studies have also investigated the potential of ADC in differentiating cyclin-dependent kinase inhibitor (CDKN2A/B) homozygous deletion in *IDH-*mutant gliomas, which is associated with a poor prognosis and assigned a higher tumor grade.^51^^,^^67^ It was reported that gliomas with non-deleted CDKN2A exhibited higher ADC values compared to heterozygous or homozygous CDKN2A deleted gliomas.^51^

Some studies have also shown the potential utility of ADC in response assessment to targeted therapies in *IDH-*mutant gliomas. In a retrospective study that assessed the changes in diffusion and perfusion metrics in *IDH-*mutant glioma patients treated with either ivosidenib or vorasidenib, a low ADC at 2-4 months from the initiation of treatment were suggestive of lower PFS. The patients with a longer PFS had relatively stable ADC, whereas a continuous decrease in ADC was seen in patients with shorter PFS.^68^ In another study that longitudinally followed an *IDH-*inhibitor (IDH305)-based phase I trial, significant associations between decreasing levels of 2-HG/Glx and increasing ADC values were found, with an increase in ADC attributed to a decrease in cellular density.^69^

Moving beyond the conventional DWI sequence, more advanced dMRI techniques such as diffusion tensor imaging (DTI), higher-order diffusion models based on advanced acquisition techniques, such as the diffusion kurtosis imaging (DKI),^70^ neurite orientation dispersion and density imaging (NODDI)^71^ and vascular, extracellular, and restricted diffusion for cytometry in tumors (VERDICT),^72^ offer deeper insights into glioma microstructure by exploring non-Gaussian diffusion and biophysical compartments. Some of these techniques have been successfully employed in differentiating *IDH-*mutant from *IDH-*wild-type gliomas. In such a study, axial kurtosis was found to be the best parameter in identifying the *IDH-*mutational status in gliomas and also had a significant correlation with Ki-67 proliferation index.^73^ NODDI is a multicompartment microstructural model that differentiates between intracellular, extracellular, and free water compartments, providing metrics such as intracellular volume fraction or neurite density index (ficvf or NDI) that may better reflect tumor cell density changes posttreatment. The ficvf, along with other DKI and DTI parameters has been used in distinguishing *IDH-*mutant from *IDH-*wild-type gliomas in a multiparametric dMRI-based study.^74^ The VERDICT is another advanced dMRI technique and computational modeling framework for simultaneous characterization of the vascular, extracellular, and intracellular components of tumors.^75^^,^^76^ These novel dMRI techniques may be used in treatment assessment in *IDH-*mutant gliomas in future studies.

A novel frontier in diffusion imaging for glioma treatment assessment is metabolite diffusion-weighted magnetic resonance spectroscopy (DW-MRS), which integrates diffusion information with metabolic profiling. By measuring metabolite diffusion properties, DW-MRS can provide insights into tumor metabolism and microstructural reorganization at a cellular level, owing to the differences in ADCs of different metabolites based on their differences in diffusion arising from the differences in their relative abundance within glial and neuronal environments and extracellular spaces.^77^ This technique may enhance the differentiation of tumor progression from treatment-related changes, especially in *IDH-*mutant gliomas where metabolic alterations are a hallmark feature. While currently limited by low SNR and acquisition challenges, future advancements in ultra-high-field MR systems and optimized acquisition schemes may position DW-MRS as a powerful tool for treatment monitoring in the era of targeted *IDH* inhibitors like vorasidenib. A pilot study showed that combining advanced dMRI and DW-MRS can noninvasively reveal microstructural and metabolic features of the tumor microenvironment, particularly in *IDH-*mutant gliomas.^78^ However, since the accurate quantification of 2-HG concentrations using existing ^1^H-MRS methods itself is quite challenging, the diffusion of this oncometabolite has not been explored to date using DW-MRS.

Although the ADC remains the most used dMRI metric for assessing treatment response in *IDH-*mutant gliomas, it provides limited specificity to underlying microstructural changes. Therefore, a multi-parametric approach by incorporating advanced dMRI metrics could potentially better assess the longitudinal effects of *IDH* inhibitors in gliomas. However, longer acquisition times, difficulties in harmonizing dMRI data across different sites and scanners, lack of resources and expertise in data acquisition and processing are some of the shortcomings that hamper the adoption of advanced dMRI techniques into the routine clinical workflows.

### Perfusion MR Imaging

Dynamic susceptibility contrast-perfusion weighted imaging (DSC-PWI) and dynamic contrast enhanced-MRI (DCE-MRI) are common perfusion MRI techniques in neuro-oncology. While DSC-PWI measures CBV, an indicator of microvascular density, DCE-MRI assesses volume transfer constant (*K*^trans^), reflecting vascular permeability and blood flow. Both serve as markers of angiogenesis and vascularity. Additional DCE-MRI parameters include tissue volume fractions of extracellular-extravascular space (ve), inversely related to cellularity, and plasma (vp), associated with tumor angiogenic activity.

The tumor vasculature of *IDH-*mutant and *IDH-*wild-type glioma blood vessels exhibit distinct features secondary to harboring discrete vascular gene expression patterns. *IDH-*wild-type gliomas demonstrate upregulation of angiogenic mediators such as angiopoietin-2 and serpin family H, promoting endothelial cell migration, extracellular matrix remodeling, and aggressive neovascular proliferation.^79^^,^^80^ In contrast, *IDH-*mutant gliomas exhibit decreased hypoxia-inducible-factor 1-α activation via the 2-HG-mediated inhibition of Egl-9 prolyl-4-hydroxylases and downstream inhibition of angiogenesis-related signaling.^81^ This molecular cascade leads to downstream suppression of vascular endothelial growth factor (VEGF)-driven angiogenic signaling, decreased microvascular proliferation, and a characteristically more organized, less permeable vascular network.

**Figure 5.**
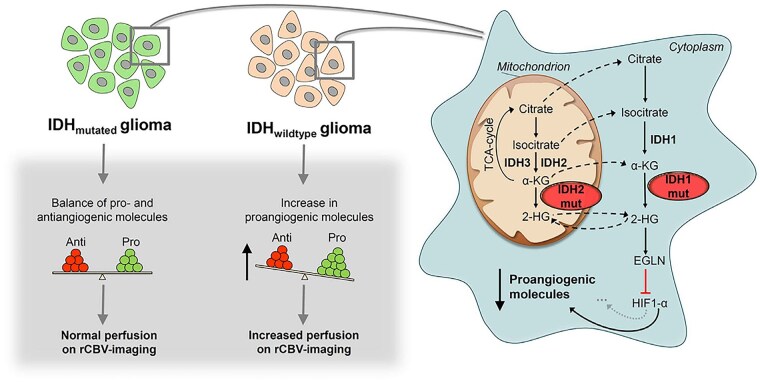
Probable mechanisms for angiogenesis and alteration in DSC-PWI-derived rCBV in *IDH-*mutant gliomas. Figure reprinted with permission from ref.^82^

These biological differences translate into consistent and measurable alterations in perfusion and permeability metrics. Multiple studies have shown that *IDH*-mutant gliomas demonstrate significantly lower rCBV than their *IDH*-wild-type counterparts, reflecting their less angiogenic phenotype.^82–84^ Additionally, histopathologic quantification shows that both intratumoral and peritumoral vessel density differ between two *IDH* genotypes, further supporting the application of perfusion-based stratification methods.^85^ Complementary permeability-sensitive techniques such as DCE-MRI also detect lower *K^trans^* and ve values in *IDH*-mutant tumors, consistent with reduced endothelial leakiness and a more intact blood–tumor barrier.^86^ These combined microvascular and permeability distinctions underpin the utility of DSC-PWI and DCE-MRI for noninvasive inference of *IDH* mutational status (Figure 5).

Building on these mechanistic insights, a meta-analysis demonstrated that the highest pooled specificity was observed for rCBV_mean_ (82%), and the highest pooled sensitivity (92%) and AUC (0.91) for rCBV 10^th^ percentile for discriminating *IDH-*mutant from *IDH-*wild-type gliomas.^87^ Another systematic review and meta-analysis reported that a rCBV_max_ threshold of 2.35 differentiated *IDH-*mutant from *IDH-*wild-type gliomas with 100% sensitivity, 61% specificity, and an AUC of 0.82.^88^ In a subgroup analysis, *IDH-*mutant with 1p19q codeletion demonstrated higher rCBV_mean_ compared to *IDH-*mutant without 1p19q codeletion.

Apart from DSC-PWI, some studies have also reported the potential utility of DCE-MRI in the identification of *IDH-*mutant gliomas.^87–90^ Using histogram analysis of DCE-MRI data, a study reported significant differences in mean, standard deviation, 90th and 95th percentile values between *IDH-*mutant and *IDH-*wild-type gliomas.^86^ Using a distributed parameter (DP) model, an advanced pharmacokinetic model for analyzing DCE-MRI data, a study found lower blood flow, vascular permeability, vp, and ve in *IDH-*mutant gliomas than those in *IDH-*wild-types. Moreover, the DP model outperformed the more commonly used Tofts model in differentiating these 2 genotypes.^89^ Some other studies have also demonstrated the potential of DCE-MRI and DWI-derived habitat imaging to characterize intratumor heterogeneity in gliomas and to serve it as a useful imaging marker in the identification of *IDH-*mutational status.^90^

Emerging evidence suggests that early, transient changes in tumor vascularity may occur during *IDH* inhibitor treatment in *IDH1*-mutant gliomas. Elevated rCBV within the first 3-6 weeks posttreatment has been associated with shorter PFS, while rCBV and the median rCBV/ADC ratio at 2-4 months serve as strong predictors of long-term PFS. The biological mechanisms underlying these changes remain unclear. It is hypothesized that the initial increase in rCBV might reflect a balance between ongoing tumor growth and therapeutic response. Additionally, the early rise in rCBV may indicate transiently increased vascularity or vascular volume. One potential explanation is that reduced 2-HG levels following *IDH* inhibition may elevate HIF-1α expression, subsequently enhancing hypoxic and proangiogenic signaling.^81^ However, after 2-4 months of treatment, these early increases in rCBV and tumor volume subside, with PFS benefits observed in patients exhibiting less pronounced rCBV elevation or stabilization.^68^  These findings suggest that the combined use of ADC and rCBV holds promise as a valuable approach for assessing the therapeutic effects of *IDH* inhibitors in gliomas.

### Amide Proton Transfer Imaging

**Figure 6.**
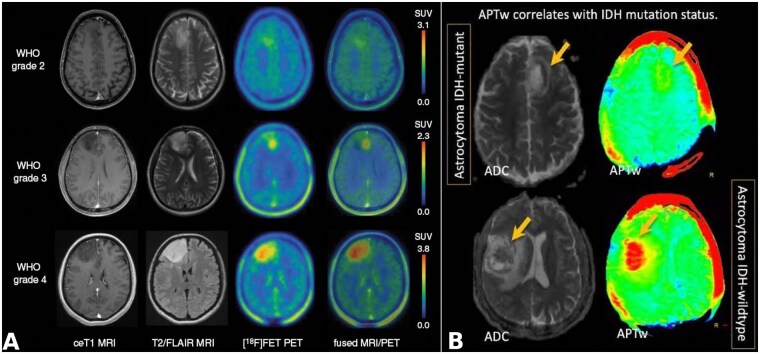
(A) Patients with CNS WHO grade 2-4 *IDH*-mutant astrocytoma (non-1p/19q codeleted): ^18^F-FET PET uptake generally correlates with tumor malignancy, regardless of contrast enhancement. (B) APTw signal in *IDH*-mutant and *IDH*-wt gliomas. Please note, higher APTw signal from *IDH*-wild type than *IDH*-mutant astrocytoma. Reprinted with permission from refs.^97^^,^^98^

Amide proton transfer imaging is a chemical exchange saturation transfer (CEST)-MRI technique that measures a reduction in bulk water intensity due to the chemical exchange of labile amide protons present within the peptide bonds of endogenous mobile proteins and peptides in tissues. Amide protons resonate at about 8.3 ppm on the MR spectrum, a chemical shift that is approximately 3.5 ppm downfield from water resonance (around 4.7 ppm). Due to the very slow exchange rate (∼30 s^−1^) of amide protons, it is possible to obtain nearly complete saturation using a low-power, long-duration saturation pulse. Given the slow exchange rate of amide protons, APT imaging can be performed even at 3T field strength and this sequence is clinically feasible and can easily be adopted in clinical settings.^91^ APT signals correlate with tissue protein-peptide concentration and exchange rate as well as tissue pH.^92^  This provides valuable insights into the tumor microenvironment, of *IDH-*mutant gliomas, which generally exhibit downregulation of peptide/proteins contents. Consequently, it is expected that the APT signal would be significantly lower in *IDH-*mutated tumors.^93^ Indeed, some studies have reported significantly higher APT signal intensities in *IDH-*wildtype gliomas compared to those in *IDH-*mutant counterparts^85^^,^^94–96^ (Figure 6). It is speculated that *IDH* gene-encoded enzymes are involved in a number of cellular functions, including amino acid metabolism, lipid metabolism, regulation of cellular redox status, and cellular epigenetics and genome-wide DNA methylation.^99^ Therefore, *IDH-*mutation is thought to cause alteration of amino acid concentrations, global transcriptional repression, and overall downregulation of mobile peptide/protein contents.^100^

### Amino-Acid-Based PET Imaging

Over the years, PET imaging has emerged as a valuable complement to conventional MR imaging in brain tumors. One of the most commonly used PET tracers to study brain tumors is the [^18^F]-2-Fluoro-2-deoxy-d-glucose ([^18^F]-FDG), with its elevated uptake reflecting increased expression of glucose transporters and/or enzymatic activity of hexokinase within neoplastic cells.^101^ However, its diagnostic potential is heavily undermined by the relatively higher uptake within the normal brain parenchyma. In the recent years, several amino acid-based PET imaging tracers have emerged as potential candidates for metabolic imaging of brain tumors.^102^ These tracers are characterized by high tumor-to-brain contrast based on their relatively high specificity for neoplastic cells and low accumulation in normal brain tissues. Their uptake occurs independently of blood-brain barrier disruption, providing a better delineation of true tumor extent, especially in non-enhancing gliomas (Figure 6).^103^ Widely used amino acid based PET tracers such as O-(2-[¹⁸F]fluoroethyl)-l-tyrosine (^18^F-FET), [¹¹C]methyl-l-methionine (¹¹C-MET), 3,4-dihydroxy-6-[^18^F]fluoro-l-phenylalanine (^18^F-FDOPA), and anti-1-amino-3-[^18^F]fluorocyclobutane-1-carboxylic acid ([^18^F]FACBC or Fluciclovine)-target energy-independent l-type amino acid transporters (subtypes LAT1 and LAT2) are frequently upregulated in brain tumors. These tracers offer a significant advantage over conventional MRI for assessment of treatment response to *IDH-*inhibitors, as they allow noninvasive in vivo visualization of active tumor cells and exhibit high correlation with tumor malignancy and grade.^97^

Radiolabeled amino acids tracers, particularly O-(2-^18^F-fluoroethyl)-l-tyrosine (^18^F-FET) are currently being used in several neuro-oncologic applications including the determination of *IDH-*mutational status in gliomas. In such a study, significantly higher static parameters [mean and maximum tumor-to-brain ratios (TBR_mean/max_)] as well as dynamic parameters [time-to-peak (TTP) and slope] were observed in *IDH-*wild-type than in *IDH-*mutant gliomas.^32^ However, the best diagnostic performance was obtained for the combination of TTP with TBR_max_ or slope with accuracies of 73%. Using ^11^C-MET PET, a related study showed an inverse correlation between tracer uptake and the presence of an *IDH-*mutation in gliomas.^104^ Dynamic amino acid PET-based studies using ^18^F-FET and ^18^F-FDOPA have also shown an association between a shorter time-to-peak curve and the *IDH*-mutational status.^105^^,^^106^ Taken together, these studies suggest that *IDH-*wild-type gliomas are associated with significantly higher tracer uptake than *IDH-*mutant gliomas.

Although widely used in Europe and gaining increasing prominence in neuro-oncology over the past decade, these amino acid tracers have failed to gain FDA clearance for diagnostic brain tumor imaging in the USA. Notably in this context, Fluciclovine, a synthetic amino acid PET tracer, has recently emerged as a promising tool in imaging of gliomas. Unlike many other tracers that rely heavily on LAT1/LAT2 transport, its cellular uptake is mediated predominantly by the neutral amino acid transporter ASCT2 (Sodium-dependent Alanine-Serine-Cysteine Transporter 2), which facilitates sodium-dependent transport of small neutral amino acids such as alanine, serine, and cysteine. In the USA, it has gained an orphan drug status for glioma imaging.^107^ Importantly, a recent prospective study demonstrated the potential utility of Fluciclovine PET in distinguishing pseudoprogression from true tumor progression in glioblastoma patients with high accuracy, making it a valuable tool for treatment planning and decision-making.^108^

Recently, the utility of amino acid-based PET has also gained considerable interest in evaluating treatment response to anti-cancer therapies in gliomas.^97^ In this direction, a preliminary study evaluated the efficacy of vorasidenib in 5 patients with residual or recurrent grade-2 gliomas (enrolled in INDIGO trial) using radiolabeled amino acid FET-PET.^109^ The investigators of this study observed significant reductions in metabolic activity as indicated by tumor-to-brain ratios (TBR_mean_ and TBR_max_) in 3 patients within 5.4-8.0 weeks of initiating vorasidenib even though no appreciable volumetric changes on T2-FLAIR images were observed. Recent studies using ^18^F-FDOPA-PET to monitor therapeutic response to *IDH*-inhibitors vorasidenib and ivosidenib have also found partial to complete responses corresponding to a reduced tracer uptake in astrocytomas and oligodendrogliomas,^110^ with effective seizure control also being reported in a case study.^111^ Collectively, these studies indicate that amino acid PET has become a promising imaging technique offering valuable insights into tumor genotypes and treatment response to targeted therapies. An innovative trajectory in glioma diagnostics is the advent of amino acid tracers designed specifically to detect *IDH*-mutations.^112^ The successful labeling of the *IDH-1* inhibitor ivosidenib with the positron emitter F-18 has been recently demonstrated with high in vivo stability.^113^ Other *IDH*-inhibitors are also being explored as potential candidates for radionuclide labeling.^114^ However, a significant difference in uptake between *IDH*-wildtype and *IDH*-mutant gliomas has not yet been achieved within these specific agents,^113^^,^^115^ warranting further research for highly selective *IDH* binding ligands for effective PET imaging in this field.

The PET RANO group advanced neuro-oncology imaging standardization by establishing the PET RANO 1.0 criteria,^116^ which provides a framework for response assessment in gliomas and brain metastases using amino acid tracers such as ^11^C-MET, ^18^F-FET, and ^18^F-FDOPA. Using these criteria, PET-positive disease is defined by a TBR threshold of 1.6 times the background SUV_mean_, while measurable disease must exceed a metabolic volume of 0.5 mL. These guidelines recommend obtaining baseline imaging within 2 weeks prior to treatment, with subsequent follow-up intervals tailored to glioma grade and specific therapeutic regimens.^117^

Future recommendations include amino acid-based PET assessment at different stages throughout the course of the disease. For instance, amino acid PET is strongly recommended to provide additional information in different scenarios like delineation of true extent of gliomas for therapy planning, differentiation of glioma recurrence from pseudoprogression, and in identifying the most malignant tumor components. Serially over the course of the disease, the recommendations ascertained high priority of amino acid PET imaging as a baseline to evaluate residual tumor at the postoperative/pre-radiotherapy stage, at the post-radiotherapy stage as a baseline for further comparison at follow-up, and also when the conventional and advanced MRI findings are inconclusive, to improve diagnosis of true progression or treatment-related changes.^118^

Nevertheless, these recent innovations and developments warrant further validation in future studies and clinical trials, including in cohorts with newly diagnosed high-grade and contrast-enhancing *IDH-*mutant gliomas, which were outside the inclusion criteria for the vorasidenib trial.^97^

## Conclusion and Future Perspectives

Future research should prioritize longitudinal validation of imaging biomarkers to monitor temporal response to targeted therapies in *IDH*-mutant gliomas, especially *IDH* inhibitors, which permit noninvasive, on-target pharmacodynamic monitoring (2-HG by ^1^H-MRS) and often induce early metabolic and microstructural changes that precede size reduction. Despite technical challenges, metabolic and physiological MRI provide robust quantifiable biomarkers that characterize the tumor microenvironment and aid diagnosis and response assessment. A multiparametric approach, integrating these methods, can provide comprehensive, reliable insights with improved diagnostic accuracy and treatment evaluation.

Although advanced MRI techniques are increasingly being integrated into routine clinical workflows, substantial variability in acquisition and analysis across centers, limits broader applicability. Standardized protocols for diffusion^119^ and perfusion MRI,^120^ should be widely adopted to improve consistency. PET offers complementary metabolic biomarkers for assessing treatment response, and integrated PET-MRI enables simultaneous, co-registered acquisition with potential gains in diagnostic accuracy, efficiency, and therapeutic monitoring.

Achieving these objectives will require coordinated efforts in data harmonization, protocol standardization, and large-scale multi-institutional validation, which will foster broader integration of advanced multiparametric imaging biomarkers into clinical neuro-oncology and ultimately improve patient outcomes.

## Data Availability

No new data were generated as a part of this review.
